# Evaluation of systemic absorption and bronchodilator effect of glycopyrronium bromide delivered by nebulizer or a dry powder inhaler in subjects with chronic obstructive pulmonary disease

**DOI:** 10.1186/s12931-019-1113-z

**Published:** 2019-06-28

**Authors:** Brian R. Leaker, Dave Singh, Grant C. Nicholson, Blanka Hezelova, Thomas Goodin, Ayca Ozol-Godfrey, Gerald Galluppi, Peter J. Barnes

**Affiliations:** 1 Respiratory Clinical Trials Ltd, 20 Queen Anne Street, London, W1G 8HU UK; 2 Medicines Evaluation Unit, Manchester University NHS Foundations Trust, University of Manchester, Manchester, UK; 3 Sunovion Pharmaceuticals Inc., Marlborough, MA USA; 4 National Heart and Lung Institute, Imperial College London, London, UK

**Keywords:** COPD, Nebulizer, Glycopyrronium bromide, Bioavailability

## Abstract

**Background:**

Effective bronchodilator therapy depends upon adequate drug deposition in the lung. COPD patients who are unable to administer medications efficiently with conventional inhalers may benefit from the use of a nebulizer device. The aim of this study was to evaluate the systemic bioavailability and bronchodilator response of glycopyrronium bromide (GLY) administered by a novel nebulizer (eFlow® closed system [CS] vibrating membrane nebulizer) or dry powder inhaler (DPI) in subjects with moderate-to-severe chronic obstructive pulmonary disease (COPD).

**Methods:**

In this randomized, open-label, single-dose, five-way crossover study, subjects received a sequence of either 50 μg GLY delivered by eFlow CS nebulizer (GLY/eFlow) or 63 μg GLY delivered by DPI (GLY/DPI), with and without activated charcoal, followed by intravenous infusion of 50 μg GLY with a washout period of 7 days between doses. Endpoints included plasma pharmacokinetics, safety and efficacy.

**Results:**

The mean (± SD) baseline predicted forced expiratory volume in 1 s (FEV_1_) of the 30 subjects who completed the study was 51 ± 15%, with a FEV_1_/forced vital capacity ratio of 50 ± 11%. Without charcoal, the absolute systemic bioavailability of GLY/eFlow and GLY/DPI were approximately 15 and 22%, respectively. Changes from baseline in FEV_1_ at 60 min post-dose, without administration of charcoal, were 0.180 L and 0.220 L for GLY/eFlow and GLY/DPI, respectively; FEV_1_ improvements were similar when charcoal was administered (0.220 L for both GLY/eFlow and GLY/DPI). There were no significant differences in spirometry between the two devices. Fewer subjects administered GLY/eFlow reported adverse events (*n* = 15) than GLY/DPI (*n* = 18).

**Conclusions:**

After single doses, GLY/DPI delivered numerically higher peak and steady state levels of drug than did GLY/eFlow. Nebulized GLY produced similar bronchodilation but lower systemic levels of drug than GLY/DPI. Slightly higher number of subjects reported adverse events with GLY/DPI than with GLY/eFlow. Nebulized GLY may offer an effective alternative to patients with COPD not adequately treated with other devices.

**Trial registration:**

NCT02512302 (ClinicalTrials.gov). Registered 28 May 2015.

**Electronic supplementary material:**

The online version of this article (10.1186/s12931-019-1113-z) contains supplementary material, which is available to authorized users.

## Background

Chronic obstructive pulmonary disease (COPD) is a prevalent respiratory disease associated with chronic lung inflammation that stems from prolonged exposure to toxic particles, primarily tobacco smoke. The persistent infiltration of inflammatory immune cells leads to parenchymal destruction and to the thickening and narrowing of small airways that result in air trapping and progressive airway obstruction. Although airway obstruction in COPD is largely irreversible, intrinsic cholinergic tone produces further narrowing that is reversible with inhaled bronchodilators. Long-acting bronchodilators, such as long-acting muscarinic antagonists (LAMA), reduce air trapping and improve symptoms in COPD patients [1].

Glycopyrronium bromide (GLY) is a competitive and potent LAMA that has a relative inhibitory effect predominantly on M_3_ muscarinic acetylcholine receptors. The binding of GLY to these receptors inhibits acetylcholine-mediated bronchoconstriction [2], reducing air trapping, increasing forced expiratory volume in 1 s (FEV_1_) and reducing symptoms.

**Fig. 1:**
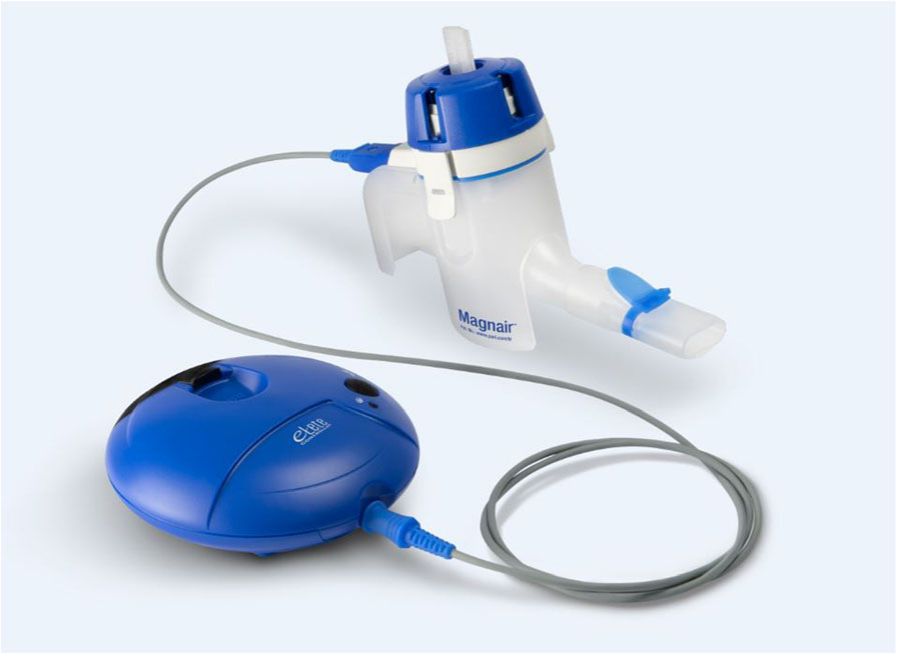
The eFlow® CS (Magnair®) nebulizer system

A soluble formulation of GLY (SUN-101, formerly known as EP-101) has been developed for nebulized delivery. Nebulizers are an effective way to deliver drugs to COPD patients who may have difficulty using handheld inhalers because of low inspiratory flow rates that may make it difficult to use dry powder inhalers (DPI) efficiently. The eFlow® closed system (CS) device (Fig. 1) is a portable, handheld, high efficiency nebulizer (PARI Pharma GmbH, Starnberg, Germany) [3]. Nebulized GLY delivered via the eFlow CS device (GLY/eFlow; Lonhala® Magnair®, 25 μg twice daily [BID]) is approved in the USA for the long-term maintenance treatment of airflow obstruction in patients with COPD [4].

The efficiency of symptom control over time greatly depends on the correct administration technique. However, studies have observed that the majority of COPD patients commit at least one error when attempting to coordinate inhalation with actuation when using pressurized metered dose inhalers (pMDI) and DPIs [5]. Patient characteristics, such as ability to generate an adequate inspiratory flow, can impact effective drug delivery to the lungs. Nebulizer devices may offer advantages to certain COPD patients who are unable to administer medications efficiently with conventional inhalers. Drug deposition in the airways of nebulized or dry powder bronchodilator formulations can be associated with the particle size generated by the respective devices. The eFlow CS nebulizer delivers GLY aerosols with a mean droplet size (3.7 μm) that may be suitable for central and peripheral airway deposition [6]. The mean size of GLY particle delivered via the Breezhaler DPI is 3.2 μm, with mean estimated intrathoracic and extrathoracic drug deposition of 31 and 57%, respectively [7].

The aim of this study was to evaluate the total systemic exposure and lung bioavailability of GLY/eFlow and GLY/DPI (Seebri® Breezhaler®) in subjects with moderate-to-severe COPD. This was achieved using treatment arms with and without activated charcoal to block gastrointestinal drug absorption, to determine the systemic pharmacokinetics (PK) of inhaled GLY versus intravenous GLY and evaluate the effects of both treatments on lung bioavailability and lung function.

## Methods

### Study subjects

Males or females aged 40 to 70 years with a clinical diagnosis of moderate-to-severe COPD were eligible. We referred to the Global Initiative for Chronic Obstructive Lung Disease (GOLD 2014) for diagnosis and severity. Subjects were required to be current or ex-smokers with at least 10 pack-year history. Women of childbearing potential were only eligible after a negative urine pregnancy test and if they agreed to use an acceptable method of birth control throughout the study. Post-bronchodilator FEV_1_ was 30–80% of predicted normal and FEV_1_/forced vital capacity (FVC) ratio of ≤0.70 at the screening visit. Subjects were required to perform reproducible spirometry to ensure accurate readings. Subjects were excluded from the study if they had concomitant clinically significant respiratory disease other than COPD, including asthma, tuberculosis, bronchiectasis or other nonspecific pulmonary disease, or respiratory tract infection within 6 weeks prior to Screening. Prior COPD medications (up to 30 days before screening; patients could use > 1 prior medication) included fluticasone/salmeterol (*n* = 11), umeclidinium/vilanterol (*n* = 1), budesonide/formoterol (*n* = 2), budesonide/eformoterol (*n* = 4), tiotropium (*n* = 11), salbutamol (*n* = 11), terbutaline (*n* = 1) and aclidinium (*n* = 1).

Subjects were fully informed of the risks of taking part in the study and were free to withdraw at any point. Written informed consent was obtained from subjects prior to any study procedure taking place. The GOLDEN 7 (SUN101–105) study protocol was approved by East of England – Cambridgeshire and Hertfordshire Research Ethics Committee (reference: 15/EE/0296) prior to patient enrollment and was conducted according to International Council for Harmonization Good Clinical Practice guidelines and the ethical principles outlined in the Declaration of Helsinki. After providing consent for study participation, subjects entered a 3-week screening period to allow for appropriate washout of their usual medication and to determine study eligibility. During the study, the use of inhaled corticosteroids (ICS) and short-acting bronchodilators was permitted.

### Study design

We performed a randomized, open-label, single-dose per dosing period, five-way crossover study in two centers in the United Kingdom. The study aimed to evaluate the PK and absolute bioavailability of GLY delivered via eFlow CS nebulizer relative to delivery via DPI. The study did not seek to directly compare the two GLY formulations and delivery devices in terms of bioequivalence.

Intravenous (IV) administration of 50 μg GLY (0.25 mL from the 200 μg/mL injection vial), which delivers 100% of the dose, was the normalization standard for PK calculations. The GLY/DPI capsules contained 63 μg GLY (approved dose in the United Kingdom), of which 88% (corresponding to 55.4 μg) is delivered through the device [8]. Therefore, a dose-normalization factor of 0.9 (50/55.4) relative to 50 μg GLY IV delivery was applied to GLY/DPI PK calculations [8]. Similar to DPIs, the efficiency of nebulizer devices depends on patient and device factors. In this study, a 50 μg dose of GLY administered via the eFlow CS nebulizer was calculated to deliver 63% of GLY (31.5 μg) during administration. A normalization factor of 1.59 (50/31.5) was applied to the GLY/eFlow PK calculations [4].

During the treatment phase of the study, subjects underwent randomization and entered one of ten treatment sequences. Five treatment arms were designed to evaluate the PK profile of GLY administered by different devices (nebulizer or DPI), with or without concomitant oral intake of activated charcoal to prevent gastrointestinal absorption (Table 1). Two 5 × 5 Latin Squares balanced for first-order carry-over effects were employed for this crossover study (Additional file 1). The data obtained were also compared to the bioavailability of GLY when administered IV. Single-dose administration was assessed as it is more sensitive than repeated dose or steady-state administration.

**Table 1:** Treatments included in the study

Treatment	Dose	Mode of administration
GLY via the eFlow CS nebulizer^a^	50 μg	Electronic nebulizer
GLY via the eFlow CS nebulizer^a^ with activated charcoal	50 μg	Electronic nebulizer
GLY via DPI^b^	63 μg	DPI
GLY via DPI^b^ with activated charcoal	63 μg	DPI
GLY sterile solution^c^	50 μg	5-min IV infusion

*CS* closed system, *DPI* dry powder inhaler, *GLY* glycopyrronium bromide, *IV* intravenous

^a^The dose for nebulized GLY was 50 μg per ampule

^b^The dose for GLY inhalation powder was 63 μg per capsule

^c^The IV dose was 50 μg (0.25 mL from the 200 μg/mL injection vial)

**Fig. 2:**
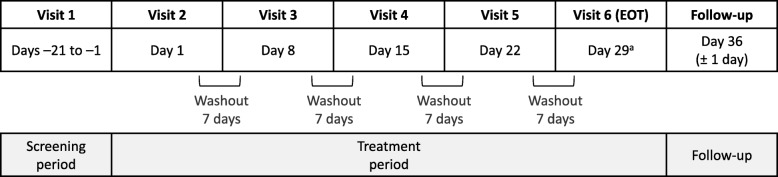
GOLDEN 7 study schematic. Washout of more than 7 days required approval by the Sponsor. All doses were administered at the same time of day (± 1 h), 7 days apart. ^a^Subjects had follow-up telephone contact on Day 36 (± 1 day) to assess safety. Subjects who discontinued prior to Visit 6 had follow-up telephone contact 5 to 7 days after their last study treatment. EOT, end of treatment

Subjects were required to attend the clinical site on the day of treatment, having fasted from solid food for 8 h prior to dosing and from fluids for 2 h prior to dosing. At each visit (Fig. 2), subjects received a single dose of the study medication according to their assigned treatment sequence. The study medication was administered in the morning and subjects remained at the study site for 24 h after dosing, during which PK samples were collected. Blood pressure and heart rate were assessed within 30 min pre-dose and 1 h (± 5 min) post-dose. A minimum 7-day washout period was required between visits. Additionally, subjects had a follow-up telephone contact on Day 36 (±1 day) to assess safety. Adverse event (AE) monitoring began once a subject signed the informed consent and continued until the last study visit.

### Pharmacokinetic assessments

The primary endpoints were maximum observed plasma concentration (C_max_) area under the plasma concentration time curve from 0 to 24 h and to infinity post-dose (AUC_0–24_ and AUC_0-∞_). Secondary endpoints included clearance (CL), fraction bioavailable (F), CL/F, volume of distribution (V_z_), V_z_/F, time to reach maximum plasma concentration (t_max_), elimination half-life (t_½_), dose normalized C_max_, and AUC_0–24_, AUC_0–48_ and AUC_0-∞_ (based on actual dose delivered).

PK samples were collected within 15 min prior to dosing, immediately at the end of inhalation (~ 5 min from the start of inhalation), immediately at the end of IV infusion (5 min from the start of IV infusion), and post-dose at 0.25–0.5 h, 1.5–2.5 h, 6–8 h, 12 h (± 15 min), 13–16 h, 24 h (± 15 min) and 48 h (± 15 min). Blood samples were collected within the prespecified time windows rather than at strict sampling times. A validated bioanalytical liquid chromatography tandem mass spectrometry method with a lower limit of quantification of 4 pg/mL was used to determine GLY concentrations.

The objective was to assess the bioavailability of GLY administered by the two devices. Oral administration of activated charcoal was added to the treatments to assess drug deposition in the oropharynx and absorption in the gastrointestinal system.

### Lung function

Spirometry was performed at 45 and 15 min pre-dose; and at 15 and 60 min post-dose during each treatment period in accordance with guidelines of the American Thoracic Society/European Respiratory Society standards 2005 [9]. Subjects receiving IV treatment were not included in these assessments. Recordings were made with a Vitalograph spirometer (Buckingham, UK). Spirometry assessments had a window of ±5 min. Subjects with a decrease in FEV_1_ of 20% or greater (based on visit pre-dose value compared with screening value) were evaluated by the investigator for continuation in the study.

### Charcoal block method

The charcoal block method can be used to determine the extent of systemic absorption via the lungs versus the gastrointestinal tract [10]. Activated charcoal (Carbomix, Beacon Pharmaceuticals, Tunbridge Wells, UK) was supplied as black granules in plastic bottles containing 81.3% activated charcoal (50 g in 61.5 g granules). Prior to use, the activated charcoal was mixed with water to form an oral suspension. A dose of 30 g was administered as a slurry in 260 mL water 2 min prior to inhalation then 5 g of activated charcoal as a slurry in 35 mL water post-dose at 2 min, and 1, 2 and 3 h. In total, 50 g of activated charcoal in a total of 400 mL water slurry was administered to each subject per charcoal arm.

### Efficacy and safety assessments

The efficacy objective of this study compared the effect of GLY delivered by the eFlow CS nebulizer with the DPI device on spirometric measurements. A secondary objective was to assess the safety of GLY inhalation administered by the eFlow CS nebulizer.

### Statistical analysis

Randomized subjects who received at least one dose of the study drug were included in the statistical analysis. All statistical procedures were performed using SAS® Version 9.2 or higher (SAS Institute Inc., Cary, NC). Measurements for spirometry, vital signs and PK parameters were analyzed using nominal visits, as applicable.

PK analyses were conducted using all subjects who were randomized, received any study drug and had evaluable PK data. Unusually high concentrations were excluded from the PK analysis as well as the descriptive statistics of concentrations. Individual subjects with AUC extrapolation greater than 20% were excluded from descriptive statistics for AUC_0-∞_, F, CL, CL/F, V_z_ and V_z_/F. Analysis of PK data was performed using WinNonlin Version 7.0 (Certara, Inc., Princeton, NJ).

A mixed-effect analysis of variance (ANOVA) was performed on the log-transformed C_max_, AUC_0–24_ and AUC_0-∞_, with terms for site, sequence, period and treatment as fixed effects. Subject nested within sequence were treated as a random effect. The results were transformed back to the original scale by exponentiation to provide geometric least-squares (LS) means and ratios, along with 90% confidence intervals (CIs). No adjustments were made for multiple comparisons. Safety endpoints were analyzed using descriptive statistics.

## Results

### Patient characteristics

A total of 30 subjects underwent randomization at two sites and 26 (87%) completed the study. A total of four patients discontinued: one lost to follow up (due to a family emergency); one requested to be withdrawn as they did not feel stable enough on the replacement inhaler; one could not commit to all overnight stays due to work commitments; and one was not brought in for Visit 2 after recruitment closed. Characteristics of the subjects at baseline are described in Table 2. All randomized subjects were included in the PK analysis. No major protocol deviations were reported. No subjects had a COPD exacerbation within 12 months prior to the study. The majority of subjects (73%) had moderate COPD and more were current smokers (57%) than former smokers.

**Table 2:** Subject characteristics at baseline

Baseline demographics	Total (*N* = 30)	
Age – Mean (SD), years	60.5 (6.6)	
Age – Range, years	40–70	
Female, n (%)	6 (20.0)	
Male, n (%)	24 (80.0)	
Active smoker, n (%)	17 (56.7)	
Former smoker, n (%)	13 (43.3)	
Number of pack-years – Mean (SD), years	42.9 (16.40)	
Number of pack-years – Range, years	14–88	
Parameter	Pre-bronchodilator	Post-bronchodilator^a^
FEV_1_ – Mean (SD), L	1.48 (0.48)	1.73 (0.41)
Pre-bronchodilator FVC – Mean (SD), L	3.27 (0.68)	3.72 (0.73)
Percent predicted FEV_1_ – Mean (SD), %	51.4 (15.08)	56.8 (12.96)
FEV_1_/FVC – Mean (SD), %	49.8 (11.12)	51.9 (12.16)

*FEV*_*1*_ forced expiratory volume in 1 s, *FVC* forced vital capacity, *SD* standard deviation

^a^Following inhalation of ipratropium bromide

### Pharmacokinetics

**Fig. 3:**
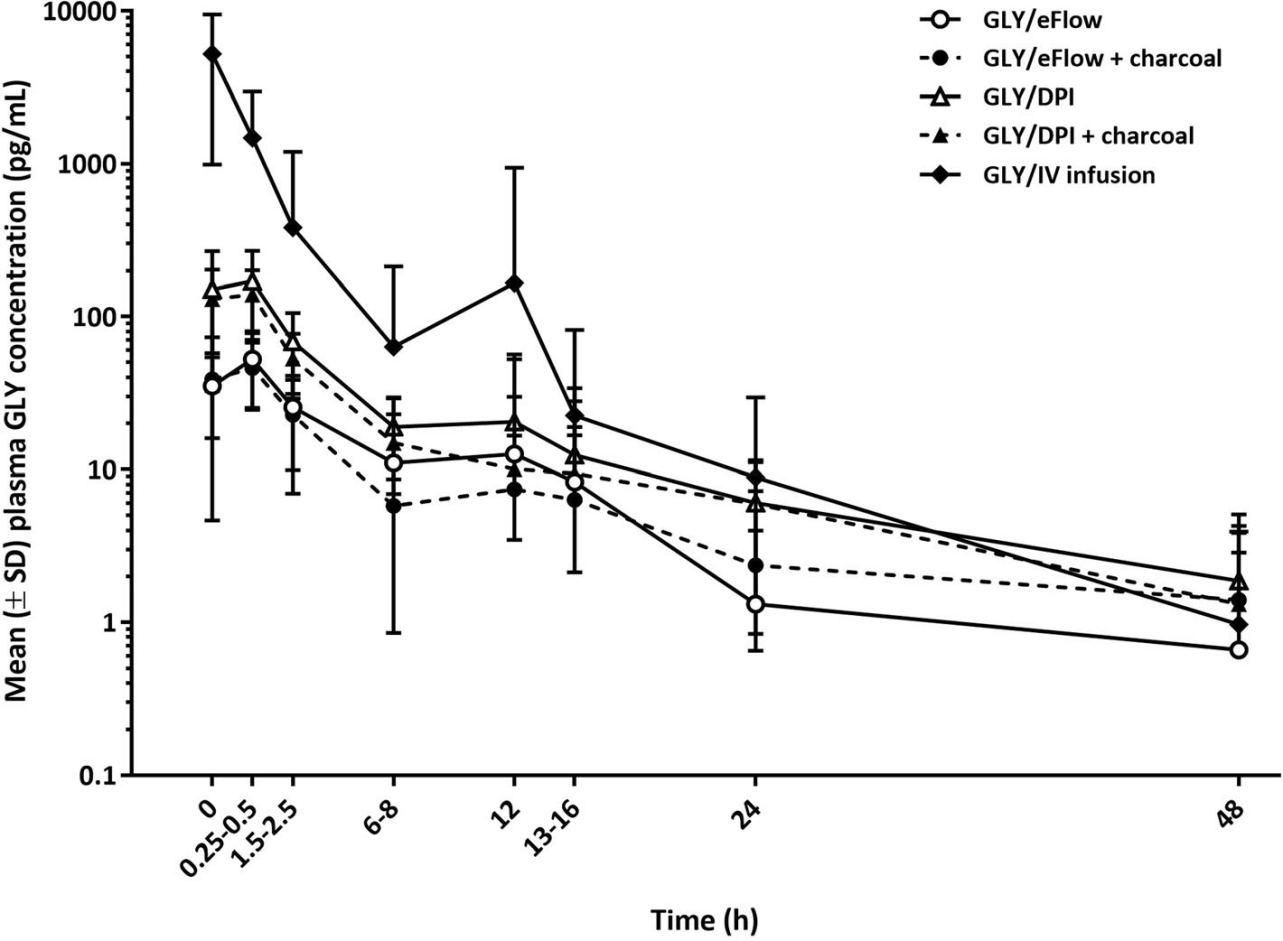
Mean (±SD) glycopyrronium bromide plasma concentration values by treatment (PK population). Note: Pre-dose was 15 min prior to dosing; 0 was immediately at the end of the inhalation or IV infusion. Unusually high concentration values of 77,400 pg/mL, 1,520,000 pg/mL, and 1,250,000 pg/mL were excluded from the PK analysis and descriptive statistics for three subjects receiving IV infusion 50 μg. Note: Lower SD bars are missing where values are below zero; negative values cannot be displayed on a logarithmic scale. DPI, dry powder inhaler. GLY, glycopyrronium bromide. IV, intravenous. PK, pharmacokinetic. SD, standard deviation

GLY plasma concentrations reached peak levels rapidly following single-dose administration (Fig. 3). As expected, peak concentration after the IV infusion was much higher, and time to reach peak plasma concentration faster than those in subjects receiving the inhaled treatments. After C_max_ was reached, plasma GLY concentrations rapidly declined. After 12 h, terminal half-life was similar among all treatments.

PK parameters are presented in Table 3. Following the single-dose administration of treatments, the C_max_ values were 200 pg/mL for GLY/DPI (dose-normalized C_max_ 180 pg/mL), GLY/eFlow 61 pg/mL (dose-normalized 98 pg/mL), GLY/DPI with charcoal 159 pg/mL (dose-normalized 143 pg/mL), GLY/eFlow with charcoal 58 pg/mL (dose-normalized 91 pg/mL) and IV infusion 5287 pg/mL. Dose-normalized AUC_0–24_ values for GLY/DPI were 523 h*pg/mL and for GLY/eFlow were 435 h*pg/mL, respectively.

**Table 3:** Summary of GLY PK parameters by treatment (PK population)

Parameter statistic	GLY/eFlow 50 μg (*N* = 29)	GLY/eFlow 50 μg + Charcoal (*N* = 29)	GLY/DPI 63 μg (*N* = 28)	GLY/DPI 63 μg + Charcoal (*N* = 26)	IV Infusion 50 μg (*N* = 27)
Cmax (pg/mL)					
N	29	29	28	26	27
Mean (SD)	61.46 (39.42)	57.51 (36.32)	199.88 (121.51)	158.96 (62.89)	5287.15 (4131.42)
CV%	64.14	63.16	60.8	39.57	78.14
tmax (h)					
N	29	29	28	26	27
Median	0.32	0.25	0.25	0.25	0.10
t½ (h)					
N	13	10	20	20	21
Median	4.66	5.08	12.99	14.20	4.69
AUC0–24 (h*pg/mL)					
N	29	29	28	26	27
Mean (SD)	273.47 (442.44)	208.20 (152.78)	581.25 (321.56)	467.56 (187.48)	3314.09 (4220.99)
CV%	161.787	73.378	55.323	40.098	127.365
AUC0–48 (h*pg/mL)					
N	29	29	28	26	27
Mean (SD)	314.90 (567.83)	255.48 (223.90)	666.06 (393.78)	546.85 (249.64)	3375.13 (4296.44)
CV%	180.32	87.64	59.12	45.65	127.30
AUC0-∞ (h*pg/mL)					
N	8	6	15	10	21
Mean (SD)	292.94 (237.34)	179.56 (75.64)	758.96 (510.22)	654.96 (347.57)	4009.65 (4722.47)
CV%	81.02	42.12	67.23	53.07	117.78
CL/F (L/h)					
N	8	6	15	10	N/A
Mean (SD)	255.12 (147.863)	316.99 (117.644)	127.82 (78.670)	131.13 (95.031)	N/A
CV%	57.957	37.113	61.546	72.473	N/A
V_z_/F (L)					
n	8	6	15	10	N/A
Mean (SD)	1316.34 (395.42)	1690.06 (286.28)	1338.96 (317.19)	1747.07 (385.31)	N/A
CV%	30.04	16.94	23.69	22.05	N/A
CL (L/h)					
n	N/A	N/A	N/A	N/A	21
Mean (SD)	N/A	N/A	N/A	N/A	28.13 (24.97)
CV%	N/A	N/A	N/A	N/A	88.78
V_z_ (L)					
n	N/A	N/A	N/A	N/A	21
Mean (SD)	N/A	N/A	N/A	N/A	156.02 (111.98)
CV%	N/A	N/A	N/A	N/A	71.77
V_ss_ (L)					
n	N/A	N/A	N/A	N/A	21
Mean (SD)	N/A	N/A	N/A	N/A	51.02 (32.57)
CV%	N/A	N/A	N/A	N/A	66.26

*AUC* area under the plasma concentration-time curve, *CL* clearance, *CL/F* apparent clearance, *C*_*max*_ maximum plasma concentration, *CV* coefficient of variation, *DPI* dry powder inhaler, *GLY* glycopyrronium bromide, *N/A* not applicable, *SD* standard deviation, *t*_*max*_ time to peak plasma concentration, *t*_*1/2*_ terminal half-life, *V*_*ss*_ volume of distribution at steady state, *V*_*z*_ volume of distribution during the elimination phase, *V*_*z*_*/F* apparent volume of distribution after extravascular dose administration

Due to variability in the PK parameters, precise quantitative comparisons of PK between dose administration with GLY/eFlow and GLY/DPI could not be performed. However, the dose-normalized PK results were sufficient to support qualitative comparisons between the two devices. Overall, the fraction of inhaled dose of GLY absorbed into plasma via the lung from the two devices was comparable. The total systemic bioavailability of GLY after dose administration from the two devices was also comparable.

**Fig. 4:**
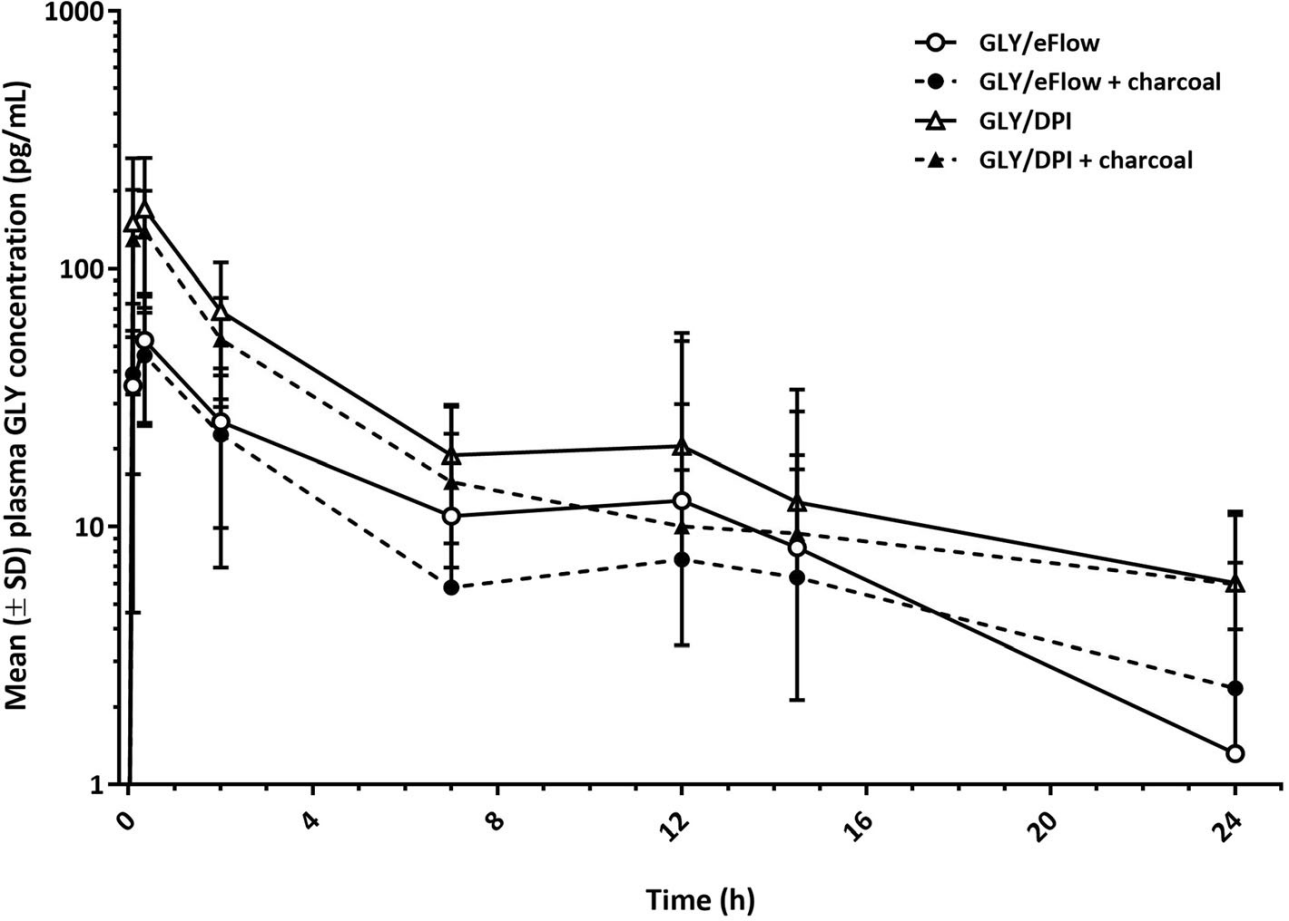
Comparison of GLY/eFlow versus GLY/DPI fully dose-normalized systemic exposure to glycopyrronium bromide. Note: Lower SD bars are missing where values are below zero; negative values cannot be displayed on a logarithmic scale. DPI, dry powder inhaler. GLY, glycopyrronium bromide. IV, intravenous. SD, standard deviation

GLY/DPI with activated charcoal treatment compared with the same inhaler with no added activated charcoal showed a geometric LS mean ratio (90% CI) for C_max_ and AUC_0–24_ of 0.91 (0.69, 1.19) and 0.92 (0.67, 1.28), respectively. The addition of activated charcoal to GLY/eFlow and GLY/DPI treatments led to an approximate 10% reduction of C_max_ and AUC_0–24_, indicating that 90% of systemic exposure was due to pulmonary absorption of both inhaled medications. The effect of the addition of oral activated charcoal on systemic bioavailability of GLY is shown in Fig. 4.

When GLY/eFlow without activated charcoal was compared with GLY/DPI without activated charcoal, the geometric LS mean ratio (90% CI) for dose-normalized C_max_ and AUC_0–24_ were 0.58 (0.44, 0.75) and 0.68 (0.49, 0.93), respectively. GLY/eFlow with activated charcoal compared with GLY/DPI with activated charcoal gave a geometric LS mean ratio (90% CI) for dose-normalized Cmax and AUC0–24 of 0.57 (0.44, 0.75) and 0.65 (0.47, 0.90), respectively. Without charcoal the absolute systemic bioavailability of GLY/eFlow was approximately 15%, while GLY/DPI showed systemic bioavailability of approximately 22%, based on dose-normalized AUC_0–24_. GLY/eFlow with activated charcoal compared with no activated charcoal treatment gave a geometric LS mean ratio (90% CI) for C_max_ and AUC_0–24_ of 0.90 (0.69, 1.17) and 0.89 (0.65, 1.22), respectively (Fig. 3). The reduction in systemic exposure of GLY with charcoal co-administration was consistent with the results for GLY/DPI from a previous study [11].

### Lung function

During treatment, spirometry data were only collected from subjects receiving inhaled medication (Table 2). Baseline mean ± SD predicted FEV_1_ was 51 ± 15% with a FEV_1_/FVC ratio of 50 ± 11%. Mean baseline pre-bronchodilator FEV_1_ was 1.48 L (SD: 0.48), while mean baseline post-bronchodilator FEV_1_ was 1.73 L (SD: 0.41). Spirometric parameters showed a mean and median improvement in all treatments at 15 min following GLY administration, with further improvement at 60 min. These findings are consistent with previous studies [3, 12].

**Fig. 5:**
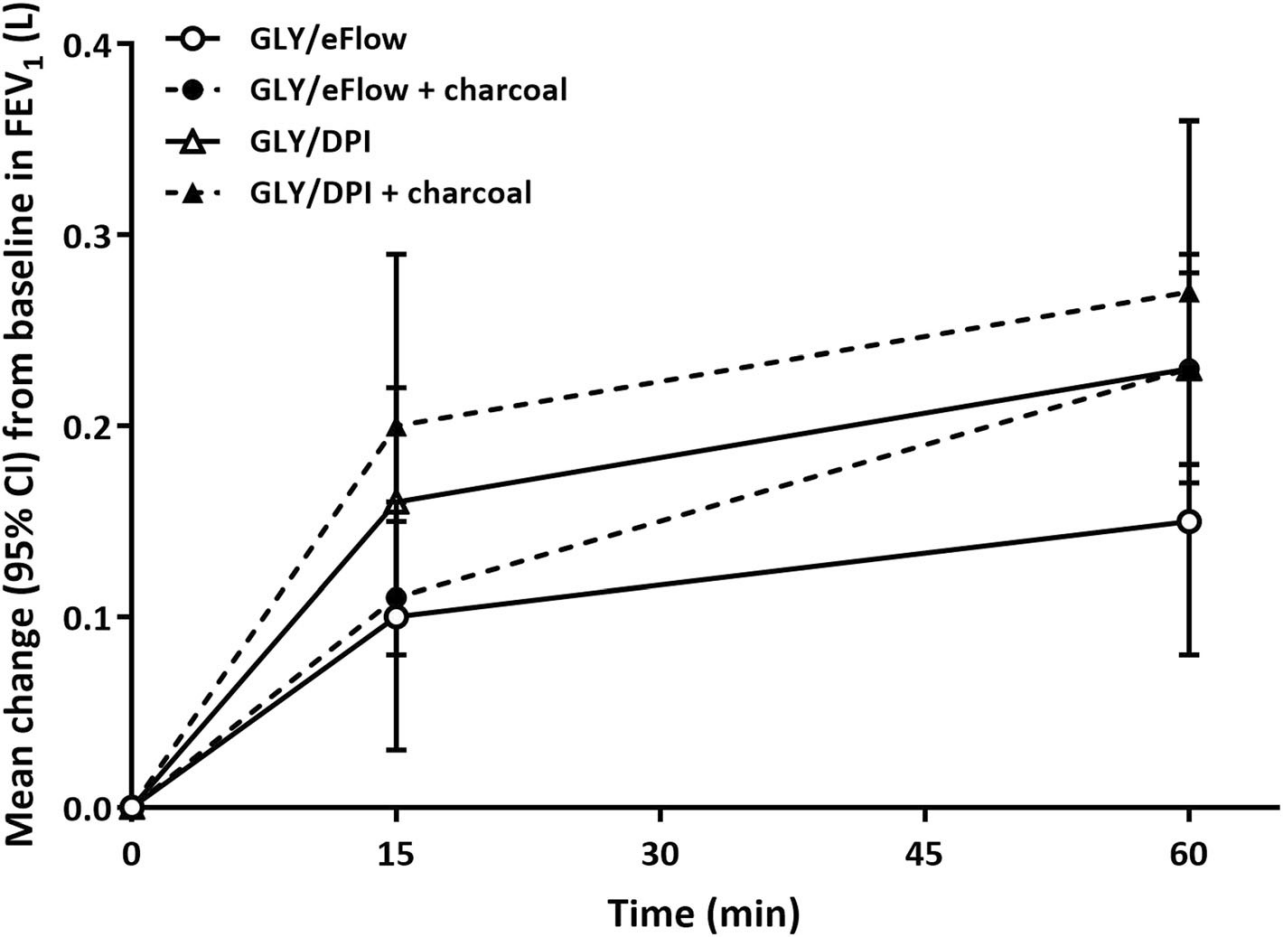
Summary of mean FEV_1_ (L) by time point on treatment day. CI, confidence interval. DPI, dry powder inhaler. FEV_1_, forced expiratory volume in 1 s. GLY, glycopyrronium bromide

Although the actual GLY delivered dose was numerically higher with GLY/DPI, than GLY/eFlow, clinically important median and mean changes from baseline in FEV_1_ were observed with both devices at 15 min after administration, with further improvements at 1 h post-dose (Fig. 5). The mean improvements in FEV_1_ showed a trend to a higher value with GLY/DPI than with GLY/eFlow, with and without charcoal (Fig. 5). Direct comparisons of FEV_1_ by treatment group were not made since the delivered doses of GLY were numerically different (GLY/DPI = 55.4 μg; GLY/eFlow = 31.5 μg).

There were no differences in the increase in FEV_1_ or FVC from baseline without charcoal (ΔFEV_1_ 15 min post-dose 0.16 ± 0.15 vs 0.095 ± 0.18 L and at 1 h post-dose 0.23 ± 0.16 vs 0.15 ± 0.20 L for GLY/DPI and GLY/eFlow without charcoal, respectively. ΔFVC at 15 min post-dose was 0.32 ± 0.37 L vs 0.21 ± 0.26 L and at 1 h post-dose, 0.44 ± 0.40 L vs 0.26 ± 0.36 L for GLY/DPI and GLY/eFlow, respectively).

### Safety evaluation

There were no deaths or serious AEs (SAEs) reported during the study. Overall, AEs were reported by 6 (20.7%) to 11 (40.7%) subjects across treatments, with a slightly higher incidence in the charcoal groups (Table 4). GLY/DPI had a slightly higher incidence of headache and dry mouth. GLY/DPI produced a higher incidence and more severe AEs of headache compared to GLY/eFlow (Table 4). Three subjects discontinued due to one or more AEs. Most AEs were assessed by the investigator as unrelated to study medication.

**Table 4:** Adverse events reported by subjects

Adverse events, n (%)	GLY/eFlow 50 μg (*N* = 29)	GLY/eFlow 50 μg + Charcoal (*N* = 29)	GLY/DPI 63 μg (*N* = 28)	GLY/DPI 63 μg + Charcoal (*N* = 27)	IV Infusion 50 μg (*N* = 27)
Any AE	6 (20.7)	9 (31.0)	7 (25.0)	11 (40.7)	7 (25.9)
Ear and labyrinth disorders	1 (3.4)	1 (3.4)	0	0	0
Cerumen impaction	1 (3.4)	0	0	0	0
Ear pain	0	1 (3.4)	0	0	0
Gastrointestinal disorders	1 (3.4)	3 (10.3)	0	3 (11.1)	0
Abdominal discomfort	0	0	0	1 (3.7)	0
Abdominal pain	0	2 (6.9)	0	0	0
Dry mouth	0	0	0	1 (3.7)	0
Nausea	0	1 (3.4)	0	0	0
Toothache	1 (3.4)	0	0	1 (3.7)	0
Vomiting	0	1 (3.4)	0	0	0
General disorders and administration site conditions	1 (3.4)	0	2 (7.1)	2 (7.4)	0
Catheter site hematoma	0	0	0	1 (3.7)	0
Catheter site pain	0	0	1 (3.6)	1 (3.7)	0
Influenza like illness	1 (3.4)	0	0	0	0
Malaise	0	0	0	1 (3.7	0
Vessel puncture site hematoma	0	0	1 (3.6)	0	0
Infections and infestations	0	2 (6.9)	1 (3.6)	0	1 (3.7)
Ear infection	0	0	1 (3.6)	0	0
Gastroenteritis	0	1 (3.4)	0	0	0
Influenza	0	0	0	0	1 (3.7)
Nasopharyngitis	0	1 (3.4)	0	0	0
Musculoskeletal and connective tissue disorders	1 (3.4)	0	2 (7.1)0	1 (3.7)0	0
Back pain	0	0	2 (7.1)	1 (3.7)	0
Musculoskeletal discomfort	1 (3.4)	0	0	0	0
Nervous system disorders	1 (3.4)	4 (13.8)	1 (3.6)	4 (14.8)	4 (14.8)
Headache	1 (3.4)	3 (10.3)	1 (3.6)	4 (14.8)	4 (14.8)
Migraine	0	1 (3.4)	0	0	0
Psychiatric disorders	0	0	0	0	1 (3.7)
Panic attack	0	0	0	0	1 (3.7)
Respiratory, thoracic and mediastinal disorders	1 (3.4)	1 (3.4)	2 (7.1)	1 (3.7)	1 (3.7)
Cough	1 (3.4)	1 (3.4)	0	0	0
Dry throat	0	0	1 (3.6)	0	1 (3.7)
Dyspnea	0	0	0	1 (3.7)	0
Oropharyngeal pain	0	0	1 (3.6)	0	0
Skin and subcutaneous tissue disorders	1 (3.4)	1 (3.4)	0	0	0
Ecchymosis	1 (3.4)	1 (3.4)	0	0	0
Vascular disorders	0	0	0	1 (3.7)	1 (3.7)
Hypertension	0	0	0	1 (3.7)	0
Phlebitis	0	0	0	0	1 (3.7)

*AE* adverse event, *DPI* dry powder inhaler, *GLY* glycopyrronium bromide

## Discussion

Glycopyrronium bromide is an effective bronchodilator in patients with COPD, and can be administered via different delivery devices. This study evaluated the systemic bioavailability and bronchodilator response of GLY treatment administered using a novel eFlow CS nebulizer or DPI. In subjects with moderate-to-severe COPD, GLY/DPI delivered numerically higher peak and steady state levels of drug compared with GLY/eFlow. Although GLY/eFlow treatment led to lower systemic levels of drug than GLY/DPI, similar, clinically important bronchodilation was produced. Overall, GLY was well tolerated regardless of delivery method, while a slightly higher number of subjects reported adverse events with GLY/DPI than with GLY/eFlow.

Clinical studies have demonstrated that inhaled GLY 50 μg monotherapy improves lung function in COPD subjects, with 24-h sustained bronchodilation [3, 13–15]. GLY/eFlow (25 and 50 μg BID) has also demonstrated statistical and clinically important improvements in lung function and health status in subjects with moderate-to-very-severe COPD [16, 17]. GLY inhalation powder can be delivered via a dry powder inhaler (Seebri® Breezhaler® [Novartis, EU]/Seebri® Neohaler® [Sunovion, USA]), dual metered dose inhaler (Bevespi Aerosphere®, AstraZeneca; Ultibro® Breezhaler® [Novartis, EU]/Utibron® Neohaler® [Sunovion, USA]) and triple combination inhaler (Trimbow®, Chiesi; Trelegy® Ellipta®, GlaxoSmithKline).

A nebulized formulation of GLY has been developed for use with the eFlow CS nebulizer and approved by the US Food and Drug Administration for the treatment of COPD patients (Lonhala® Magnair®, 25 μg BID, Sunovion Pharmaceuticals Inc.) [4]. The eFlow CS device is an electronic nebulizer system, which utilizes a vibrating membrane to aerosolize the drug formulation for administration during normal tidal breathing.

This study evaluated the total systemic exposure and lung bioavailability of GLY/eFlow and GLY/DPI administered with and without activated charcoal in subjects with moderate-to-severe COPD. Plasma concentrations were measured with and without concomitant administration of activated charcoal, which was compared with IV GLY.

The PK observations presented here are consistent with data reported previously, where it was shown that GLY was detectable in the circulation after inhalation (t_max_ values of less than 20 min), and plasma concentration decreased to less than 10% at 12 h post-inhalation [3, 18]. As expected, the peak concentration with the IV infusion was much higher, and time to reach peak plasma concentration was faster than the other treatments. After C_max_ was reached, plasma GLY concentrations rapidly declined. After the 12-h post-administration time point, t_1/2_ was similar among all treatments.

Our results are comparable with those of a population PK analysis conducted using GLY concentration-time data from four clinical studies involving 117 patients with moderate-to-severe COPD (Sunovion, data on file). As assessed by the population PK analysis, there was no effect of age, gender, race, weight and COPD severity on the PK of GLY/eFlow. Model-based diagnostics suggested that the exposure to GLY/eFlow increased with dose in a linear manner up to 400 μg. The mean apparent total systemic clearance (CL/F) for a typical patient was 206 L/h (coefficient of variation [CV%], 28%).

The peak GLY plasma concentration after dosing was numerically lower in the GLY/eFlow treatment arm compared to the GLY/DPI treatment arm, which is likely due to the numerically lower delivered dose. However, from 2 h post-dose there was no difference in plasma concentration between the two treatments. The absolute bioavailability of GLY/eFlow is estimated to be approximately 15%, and the absolute bioavailability of GLY/DPI is estimated to be approximately 22%.

AUC measurements give an indication of total systemic exposure via oral and pulmonary routes for compounds that do not have an extensive first pass effect. By administering charcoal 5 min prior to dosing with inhaled medication, non-pulmonary absorption is substantially reduced, therefore giving an indication of total pulmonary exposure. This study reports that approximately 90% of systemic exposure to GLY was from GLY deposited in the airways and only 10% was from GLY absorption in the oropharynx and gastrointestinal system. This is indicated by the reduction of both C_max_ and AUC_0–24_ with charcoal co-administration, as compared with GLY administered without charcoal. A previous study assessing systemic exposure and lung deposition of different delivery methods using the charcoal-block method confirmed that AUC is a suitable measurement of lung bioavailability [19].

GLY/DPI delivered approximately double the AUC_0–24_ levels of GLY than GLY/eFlow after single-dose administration. It should be emphasized that GLY/DPI delivered a higher absolute dose (55.4 μg) than GLY/eFlow (31.5 μg). However, the increase in systemic PK values for GLY/DPI may also reflect greater efficiency of drug delivery. The higher peak levels may account for the marginally higher adverse events recorded for GLY/DPI. There was no substantial difference in spirometry measurements between GLY/DPI and GLY/eFlow. GLY/eFlow produced similar bronchodilation but lower systemic levels of drug with no major absorption through the upper gastrointestinal tract, as demonstrated by the charcoal-block method.

We observed clinically relevant improvements in lung function shortly after drug administration, which further improved for at least 45 min. In general, the median changes in FEV_1_ from baseline values were numerically higher for both drug formulations without charcoal administration. A direct comparison of FEV_1_ was not made since the delivered doses of GLY were numerically different (GLY/DPI = 55.4 μg; GLY/eFlow = 31.5 μg). The median improvement in FEV_1_ at 1 h post-dose for GLY/DPI with or without charcoal was 0.220 L, whereas for GLY/eFlow with or without charcoal was 0.220 L and 0.180 L, respectively. This study showed that both GLY formulations have clinically meaningful benefit in the selected study population [20].

GLY/eFlow was well tolerated. There were no deaths or SAEs. The AE profile in this study was consistent with those reported in larger studies. The incidence and severity of anticholinergic AEs were numerically higher in GLY/DPI compared to GLY/eFlow and higher in both charcoal groups. This AE profile is likely to correlate with the higher C_max_ seen in the GLY/DPI group that occurred immediately after drug administration.

It is important to note that subjects in this study were appropriately trained to use the DPI device. In real-life settings, this is unfortunately not the case; several studies have shown that patients, especially the elderly cohorts, commit at least one error when using their devices, mostly associated with coordination and actuation [5, 21–23].

While lung function tests and exercise ability are a reliable aspect of the assessment of treatment for obstructive lung diseases, other factors, such as patient experience and quality of life should be taken into consideration when treatment recommendations are made. A relevant aspect of determining the appropriate device for a patient is their preference, which also may be related to a better quality of life due to improved treatment satisfaction. For patients who prefer using nebulizers instead of inhalers [24], the eFlow CS device could be an alternative treatment option.

## Conclusion

The results of this study show that charcoal reduced plasma GLY AUC_0–24_ by approximately 10% for both GLY/eFlow and GLY/DPI, suggesting most of the plasma exposure to GLY was via pulmonary absorption, irrespective of inhalation device. The absolute systemic bioavailability of GLY was approximately 15% with the eFlow CS nebulizer and approximately 22% with the DPI device. Given the variability observed historically and in this study, these PK values are considered to be similar and do not differ substantially from one another. GLY/eFlow was generally well tolerated. All single doses of inhaled study drug resulted in clinically important improvements in lung function and no PK/pharmacodynamic relationship was identified.

## Additional file

Additional file 1: Sequence of treatments. (DOCX 33 kb)

## Data Availability

Sunovion Pharmaceuticals Inc. is part of a clinical trial data sharing consortium that facilitates access for qualified researchers to selected anonymized clinical trial data. For up-to-date information on data availability please visit https://www.clinicalstudydatarequest.com/Study-Sponsors.aspx and click on Sunovion.

## Abbreviations

AE: Adverse events
AUC: Area under the plasma concentration time curve
BID: Twice daily
CI: Confidence interval
CL: Clearance
C_max_: Maximum observed plasma concentration
COPD: Chronic obstructive pulmonary disease
CS: Closed system
CV%: Coefficient of variation
DPI: Dry powder inhaler
F: Fraction bioavailable
FEV_1_: Forced expiratory volume in 1 s
FVC: Forced vital capacity
GLY: Glycopyrronium bromide
GOLD: Global Initiative for Chronic Obstructive Lung Disease
ICS: Inhaled corticosteroid
IV: Intravenous
LAMA: Long-acting muscarinic antagonist
LS: Least squares
PK: Pharmacokinetics
pMDI: Pressurized metered dose inhaler
SAE: Serious adverse event
SD: Standard deviation
t_½_: Elimination half-life
t_max_: Time to reach maximum plasma concentration
V_ss_: Volume of distribution at steady state
V_z_: Volume of distribution
