# Supplementary material for: Evaluation of systemic absorption and bronchodilator effect of glycopyrronium bromide delivered by nebulizer or a dry powder inhaler in subjects with chronic obstructive pulmonary disease
Source: Respir Res. 2019 Jun 28;20:132. doi: 10.1186/s12931-019-1113-z (PMC6599298; doi:10.1186/s12931-019-1113-z)
Supplement: Supplementary file 1 — Sequence of treatments. (DOCX 33 kb) [file 12931_2019_1113_MOESM1_ESM.docx]

**Additional file 2.** Sequence of treatments

| **Planned sequence** (n randomized subjects) | **Treatment period** | | | | |
| --- | --- | --- | --- | --- | --- |
|  | **Week 1** | **Week 2** | **Week 3** | **Week 4** | **Week 5** |
| **A** (3) | GLY/DPI+ | GLY/eFlow+ | IV GLY | GLY/eFlow | GLY/DPI |
| **B** (3) | GLY/eFlow | GLY/eFlow+ | GLY/DPI | GLY/DPI+ | IV GLY |
| **C** (4) | GLY/eFlow | GLY/DPI | GLY/eFlow+ | IV GLY | GLY/DPI+ |
| **D** (3) | IV GLY | GLY/DPI+ | GLY/DPI | GLY/eFlow+ | GLY/eFlow |
| **E** (3) | GLY/DPI | IV GLY | GLY/eFlow | GLY/DPI+ | GLY/eFlow+ |
| **F** (3) | GLY/DPI+ | IV GLY | GLY/eFlow+ | GLY/DPI | GLY/eFlow |
| **G** (2) | GLY/DPI | GLY/eFlow | IV GLY | GLY/eFlow+ | GLY/DPI+ |
| **H** (3) | GLY/eFlow+ | GLY/eFlow | GLY/DPI+ | GLY/DPI | IV GLY |
| **I** (3) | IV GLY | GLY/DPI | GLY/DPI+ | GLY/eFlow | GLY/eFlow+ |
| **J** (3) | GLY/eFlow+ | GLY/DPI+ | GLY/eFlow | IV GLY | GLY/DPI |

+, with activated charcoal. DPI, dry powder inhaler. GLY, glycopyrronium bromide. IV, intravenous.
